# The Delta-Opioid Receptor; a Target for the Treatment of Pain

**DOI:** 10.3389/fnmol.2020.00052

**Published:** 2020-05-05

**Authors:** Béatrice Quirion, Francis Bergeron, Véronique Blais, Louis Gendron

**Affiliations:** Faculté de Médecine et des Sciences de la Santé, Département de Pharmacologie-Physiologie, Institut de Pharmacologie de Sherbrooke, Centre de Recherche du Centre Hospitalier Universitaire de Sherbrooke, Université de Sherbrooke, Sherbrooke, QC, Canada

**Keywords:** delta-opioid receptor, pain, primary afferents, G protein-coupled receptors, trafficking

## Abstract

Nowadays, pain represents one of the most important societal burdens. Current treatments are, however, too often ineffective and/or accompanied by debilitating unwanted effects for patients dealing with chronic pain. Indeed, the prototypical opioid morphine, as many other strong analgesics, shows harmful unwanted effects including respiratory depression and constipation, and also produces tolerance, physical dependence, and addiction. The urgency to develop novel treatments against pain while minimizing adverse effects is therefore crucial. Over the years, the delta-opioid receptor (DOP) has emerged as a promising target for the development of new pain therapies. Indeed, targeting DOP to treat chronic pain represents a timely alternative to existing drugs, given the weak unwanted effects spectrum of DOP agonists. Here, we review the current knowledge supporting a role for DOP and its agonists for the treatment of pain. More specifically, we will focus on the cellular and subcellular localization of DOP in the nervous system. We will also discuss in further detail the molecular and cellular mechanisms involved in controlling the cellular trafficking of DOP, known to differ significantly from most G protein-coupled receptors. This review article will allow a better understanding of how DOP represents a promising target to develop new treatments for pain management as well as where we stand as of our ability to control its cellular trafficking and cell surface expression.

Affecting more than one-third of the North American population during their lifetime, chronic pain is more frequent than cardiovascular diseases, diabetes, and cancer combined (Gaskin and Richard, 2012). According to the 2010 Medical Expenditure Panel Survey (MEPS), chronic pain is one of the most important socio-economic burdens in the U.S., with estimated annual costs ranging from $560 to $635 billion; $261 to $300 billion in direct health care costs and $299 to $335 billion in lost productivity and other indirect costs (Gaskin and Richard, 2012). With the aging population, these numbers are predicted to double within the next decade. Despite notorious adverse effects and lack of effectiveness in many types of pain, opioids remain the standard of care for treating moderate to severe conditions (Ballantyne et al., 2016). The use of opioids has led to their widespread diversion and misuse calling upon the U.S. Department of Health and Human Services (HHS) to declare an opioid crisis in 2017^1^ (Kirson et al., 2017; Iwanicki et al., 2018). In concert with pharmaceutical companies and academic research centers, three main goals were elaborated: (1) design safe, effective, and non-addictive strategies to manage chronic pain; (2) new, innovative medications and technologies to treat opioid use disorders; and (3) improved overdose prevention and reversal interventions to save lives and support recovery^2^.

Opioids act *via* the opioid receptors, namely Mu (μ), Kappa (κ), and Delta (δ). The most prescribed opioids (e.g., morphine, Fentanyl, codeine) preferentially target the μ opioid receptors (MOP). These substances, being among the most potent analgesics, produce diverse effects and are responsible for almost all prototypic opioid unwanted effects such as euphoria, mental clouding, sedation, respiratory depression and cough suppression, pupillary miosis (oculomotor nerve parasympathetic stimulation), antidiuresis, urinary retention, nausea and vomiting, bradycardia and vasodilation, constipation and biliary retention, and histamine release (Katzung et al., 2009; Khademi et al., 2016).

Selective activation of the δ opioid receptor (DOP) has great potential for the treatment of chronic pain (Kieffer and Gavériaux-Ruff, 2002; Gavériaux-Ruff and Kieffer, 2011) with ancillary anxiolytic- and antidepressant-like effects (Chu Sin Chung and Kieffer, 2013). Their ability to cause emotional responses is highly desirable because of the frequent association of anxiety and mood disorders with chronic pain (Goldenberg, 2010a, b). Compared to MOP agonists, molecules acting on DOP typically show reduced adverse effects. Here, we review important findings supporting a role for DOP in the treatment of chronic pain. Because its physiological roles are directly related to its cellular and subcellular expression, we also discuss the distribution of DOP along the pain pathways as well as the cellular mechanisms regulating its trafficking to the cell surface.

## Ascending and Descending Pain Pathways

Pain processing runs through a distinctive neurological pathway. The propagation of pain starts with the activation of receptors, called nociceptors, which are found widely in peripheral tissues, muscles, and organs (Almeida et al., 2004). The nociceptive sensory fibers transform stimuli and generate a membrane potential which, if the threshold is reached, generates an impulse (Khalid and Tubbs, 2017). Whether or not the action potential is initiated depends on the intensity of the stimulus (Mense, 1983; Millan, 1999; Bester et al., 2000; Almeida et al., 2004). Nociceptors have a high threshold compared to other receptors and only a strong, potentially harmful stimulus, activates them (Woolf and Ma, 2007). The impulse propagates along the primary afferent fiber to reach the central nervous system. Primary afferent fibers are pseudo-unipolar neurons which means their cell body has one emerging axon that divides in peripheral and central projections. The peripheral branch innervates the target organ (skin, muscle, viscera) while the central axon projects to the dorsal horn of the spinal cord which is organized in anatomically different laminae (Basbaum and Jessell, 2000; Almeida et al., 2004; Khalid and Tubbs, 2017). The cell bodies of the primary afferents are located in dorsal root (DRGs) and trigeminal ganglia (TGs; Basbaum et al., 2009; Dubin and Patapoutian, 2010). These neurons are commonly classified according to their size (small, medium and large diameter neurons), conducting velocity, and levels of myelination. Interestingly, DRG and TG neurons have various roles when it comes to proprioception, exteroception, and nociception. Medium diameter myelinated (Aδ) fibers and small diameter unmyelinated C fibers are mainly responsible for nociception (Mense, 1983; Woolf and Ma, 2007; Garland, 2012). Aα and Aβ fibers are also primary afferent fibers respectively implicated in proprioception and touch, although they may also be involved in nociception (Watson, 1981; Djouhri and Lawson, 2004; Abraira and Ginty, 2013; Le Pichon and Chesler, 2014). After the integration of the noxious stimuli at the spinal cord level, the nociceptive signal travels through different ascending pathways to the thalamus and the brainstem, namely the spinothalamic and the spinoreticulothalamic tracts (for a review see Almeida et al., 2004). Once the signal reaches the cortical structures, it is processed at the level of the sensory, the cingulate, and the insular cortices (Apkarian et al., 2005; Basbaum and Julius, 2006). In addition to the ascending pain pathways, an endogenous inhibitory system called the descending pain modulatory circuit is also part of the pain circuitry. This circuit involves multiple regions of the central nervous system such as the frontal neocortex, the hypothalamus, the amygdala, the rostral anterior cingulate cortex (rAAC), the periaqueductal gray region (PAG), the medulla and the rostroventral medulla (RVM; Fields et al., 2006; Ossipov et al., 2010). This ensemble projects to the dorsal horn of the spinal cord to modulate the ascending pain signal (Fields et al., 2006; Ossipov et al., 2010). In order to alleviate pain, a drug must therefore act on a target expressed at least in one of these structures.

## Delta Opioid Receptor Distribution in the Central Nervous System

To this day, the cellular distribution of DOP along the pain pathways remains unclear. Depending on the technique used to assess the expression of DOP in tissue, significant differences are observed concerning its localization. Approaches such as *in situ* hybridization, immunohistochemistry, autoradiography using radiolabeled ligands, GTPγS assay and genetically modified mouse models have been used to study the istribution of DOP (Quirion et al., 1983; McLean et al., 1986; Mansour et al., 1987, 1994; Dado et al., 1993; Simonin et al., 1994; Cahill et al., 2001a; Mennicken et al., 2003; Guan et al., 2005; Scherrer et al., 2009; Wang et al., 2010). Small discrepancies in the distribution of the receptor between studies can be attributed to differences in tissue processing, the use of different species, and/or ligand sensitivity. However, one of the most important controversies in the field arises from a comparison of the receptor distribution using antibodies raised against DOP and the use of DOP-eGFP knockin (DOP-eGFP KI) transgenic mice. The distribution of DOP using these two techniques is noticeably different and certain concerns arise for either one. Admittedly, most antibodies raised against DOP lack specificity since many commercially available and custom antibodies stain the spinal cord of DOP-KO mice similarly to the wild type mice (Scherrer et al., 2009). Although the use of mice expressing chimeric receptors bearing a 23 kDa GFP protein within their intracellular loops or their C-terminal tail might not be the best approach to visualize the endogenous distribution of DOP, this tool offers the possibility to directly detect the receptor in native or fixed tissues, sometimes without the need to use antibodies. However, the use of such intracellular tags (like GFP) is a matter of debate. Indeed, the cellular distribution and compartmentalization of DOP (Gendron et al., 2015; Zhang et al., 2015) and other GPCRs (McLean and Milligan, 2000; Madziva and Edwardson, 2001; McDonald et al., 2007) is altered by the addition of an eGFP tag. To summarize, each technique has its strengths and weaknesses for identifying DOP in tissues.

### Expression of DOP in the Brain

The delta-opioid receptor (DOP) is widely distributed in the brain, without significant difference between rodents and humans (Simonin et al., 1994). Along the pain pathways, DOP is expressed in structures of both the ascending and the descending pain pathways. More specifically, DOP is found in the PAG, the RVM, the cerebral cortex and the amygdala (Mansour et al., 1987, 1994, 1995; Tempel and Zukin, 1987; Sharif and Hughes, 1989; Kiefel et al., 1993; Slowe et al., 1999; Cahill et al., 2001a; Peng et al., 2012). More interestingly, DOP likely participate not solely in the control of pain but also of mood disorders such as anxiety and depression (Chu Sin Chung and Kieffer, 2013; Lutz and Kieffer, 2013).

### Expression of DOP in the Spinal Cord

In the rodent spinal cord, the expression of DOP is predominant in superficial laminae I and II. The expression also extends to other laminae, including a broad distribution throughout the gray matter (Cahill et al., 2001a,b, 2003; Mennicken et al., 2003) and motoneurons located in the ventral horns (Wang et al., 2018). Various techniques including immunohistochemistry (Cahill et al., 2001a), autoradiography using DOP-selective radioligands (Mennicken et al., 2003; Bardoni et al., 2014; Wang et al., 2018), *in situ* hybridization (Cahill et al., 2001a; Mennicken et al., 2003; Wang et al., 2018), and transgenic mice (DOP-eGFP KI mice; Scherrer et al., 2009) supports this wide distribution of DOP. The study of the cellular distribution of DOP in DOP-eGFP KI mice revealed that most DOP-positive neurons located in lamina II express TLX3, a marker for spinal excitatory interneurons. The presence of DOP in these neurons is supported by electrophysiological studies where the resting membrane potential and action potential firing patterns were measured (Wang et al., 2018). These neurons are now known to be somatostatin-positive neurons likely involved in the transmission of mechanical noxious stimuli (Bardoni et al., 2014; Chamessian et al., 2018; Wang et al., 2018).

In higher species, the distribution of DOP in the spinal cord looks slightly different (Mennicken et al., 2003). In monkeys, radioligand binding revealed that DOP is expressed at a higher level in superficial rather than in deeper laminae, with a high density of DOP labeling in lamina II (Honda and Arvidsson, 1995; Zhang et al., 1998; Mennicken et al., 2003). More interestingly, DOP binding sites in the human spinal cord are even more restricted to the superficial laminae, with no apparent labeling in laminae III-X (Mennicken et al., 2003). A more restricted expression of DOP in the human spinal cord calls for the role of DOP in the regulation of the activity of primary afferents. The apparent lack of DOP mRNA in the human spinal cord (Peckys and Landwehrmeyer, 1999; Mennicken et al., 2003) suggests that the receptor is present on synaptic terminals of primary afferents projecting to laminae I and II while, in rodents and monkeys, *in situ* hybridization showed a widespread distribution of DOP mRNA throughout the gray matter. DOP mRNA is also expressed in motoneurons in the ventral horn of the mouse, rat, and monkey spinal cord, but not in humans (Mennicken et al., 2003). To summarize, the expression pattern of DOP mRNA and protein in the human spinal cord, but also other species, supports a role for DOP in pain control. In all species, but more specifically in humans, the presence of DOP on what appears to be the primary afferent endings suggests that this receptor is synthesized in primary afferents and transported to the spinal cord (Mennicken et al., 2003). This is further supported by the fact that deafferentation significantly decreased the density of DOP in the dorsal horn of the spinal cord in rodents (Dado et al., 1993).

### Expression of DOP in Primary Afferents

As mentioned above, DOP is expressed in primary afferents, the very first step of pain processing pathways. Somatosensory neurons, more specifically DRG neurons, are responsible for the detection and the transmission of noxious stimuli to the brain. In DRG neurons, opioid receptors regulate cell excitability and neurotransmitter release (François and Scherrer, 2018). However, the exact distribution of DOP in these neurons remains controversial.

There are two diverging opinions on DOP’s distribution in somatosensory neurons. The first school of thought infers that DOP is found mainly in large myelinated DRG neurons and reports a low level of co-expression with MOP (Scherrer et al., 2009; Bardoni et al., 2014). The DOP-eGFP KI mouse model reveals that DOP labeling is mainly observed in NF200-positive cells, a marker of myelinated DRG neurons (Scherrer et al., 2009). Single-cell RNA sequencing further supports that *Orpd1* transcripts were solely found in NF200-positive DRG neurons (Usoskin et al., 2015). Moreover, most DOP-eGFP-positive cells also express TRPV2, a channel found in myelinated neurons. Since most myelinated somatosensory neurons are mechanosensitive, this distribution suggests that DOP plays a role in mechanical pain (Usoskin et al., 2015). In support of this hypothesis, a significant level of DOP labeling is found in high-threshold mechanosensitive A-fibers (A HTMRs). Interestingly, DOP-eGFP is also co-expressed with Ret and/or TrkC, markers of low-threshold mechanosensitive A-fibers (A LTMRs; François and Scherrer, 2018). Although in small proportion, it is worth noting that DOP-eGFP is also found in unmyelinated DRG neurons. These neurons were mainly identified as small unmyelinated neurons expressing IB4 and P2X3, markers for nonpeptidergic C nociceptors. Only a few DOP-eGFP-positive neurons, if any, were identified as SP-, CGRP- and TRPV1-positive, confirming that DOP is rarely found in small peptidergic DRG neurons. Using an antibody raised against MOP, immunohistochemical studies revealed that in DOP-eGFP KI mice, DOP and MOP were rarely co-expressed in the same DRG neurons (less than 5% of co-expression) but rather segregated in distinct populations, suggesting that these receptors might play different roles when it comes to pain modulation. Indeed, DOP was shown to be mostly implicated in mechanical pain control while MOP controls thermal pain (Scherrer et al., 2009).

The second school of thought rather promotes the idea that the distribution of DOP includes small DRG neurons (Ji et al., 1995; Wang and Wessendorf, 2001) and that DOP and MOP are co-expressed in some neurons. Immunohistochemistry, RT-PCR and single-cell PCR techniques have shown that DOP-positive DRG neurons were often of small-diameter (Ji et al., 1995; Guan et al., 2005; Wang et al., 2010), with one-third of these neurons also co-expressing SP or CGRP (Guan et al., 2005; He et al., 2010; Wang et al., 2010). Additionally, some studies suggested that DOP is found both in small and large DRG neurons. Indeed, RT-PCR, *in situ* hybridization and immunohistochemistry approaches revealed that DOP is located almost equally in all types of neurons (Wang and Wessendorf, 2001; Mennicken et al., 2003; Rau et al., 2005). The presence of DOP on both myelinated and unmyelinated peptidergic and non-peptidergic somatosensory neurons supports a role in heat and mechanical pain control. In animal models, the selective activation of DOP was indeed shown to alleviate both mechanical and thermal pain (Tseng et al., 1997; Gendron et al., 2007b; Holdridge and Cahill, 2007; Beaudry et al., 2009; Dubois and Gendron, 2010; Otis et al., 2011; Normandin et al., 2013). Among these findings, our group has shown, using *in vivo* electrophysiology, that the activation of spinal DOP with deltorphin II led to the inhibition of the diffuse noxious inhibitory controls (DNIC) activated by heat and mechanical noxious stimuli (Normandin et al., 2013). We further observed that both DOP and MOP were able to inhibit the noxious heat- and mechanical-induced release of SP in the spinal cord (Beaudry et al., 2011; Normandin et al., 2013).

A thorough characterization of DOP on central terminals of primary afferents revealed important differences between rodents and primates. When compared to rodents, not only more DRG neurons express DOP, the receptor is also expressed in a higher proportion of medium- and small-diameter neurons in human and non-human primates, supporting a more specialized role for DOP in pain processing in higher species (Mennicken et al., 2003).

Regardless of the subcellular distribution of DOP in nociceptors, electrophysiological studies confirmed that the activation of DOP reduced the amplitude of evoked excitatory postsynaptic currents (Glaum et al., 1994). This suggests that the excitatory glutamatergic transmission in the lamina II of the spinal cord is inhibited by a presynaptic action of DOP (Glaum et al., 1994; Wang et al., 2010; François and Scherrer, 2018), highlighting a role for the receptor in pain modulation at the presynaptic level. If the distribution of DOP in primary afferents and spinal cord neurons remains a matter of debate, its expression in all structures involved in pain processing, as well as its more restricted expression within structures implicated in pain modulation in higher species, raise DOP among the most promising targets for the development of novel pain therapies.

## Trafficking of DOP in Neurons

The major challenge in making new therapeutics for the treatment of pain, and most particularly chronic pain, is to develop molecules maximizing analgesia while minimizing adverse effects. Because they do not produce the common adverse effects associated with clinically used opioids, this is exactly where DOP-selective agonists could be useful (Pradhan et al., 2011). However, under normal conditions, DOP agonists only have weak analgesic effects in animal models of evoked pain. It is now recognized that the weak analgesic potency of DOP agonists is the consequence of a low level of expression at the plasma membrane (Cahill et al., 2007; Pradhan et al., 2011). Indeed, several studies employing electron microscopy immunogold labeling, photoaffinity-labeling of endogenous receptors, and biochemical subcellular fractionation techniques showed that DOP mainly localizes in intracellular compartments and organelles while only a small portion is associated with the plasma membrane of neurons (Pasquini et al., 1992; Zerari et al., 1994; Arvidsson et al., 1995; Cheng et al., 1995, 1997; Elde et al., 1995; Zhang et al., 1998; Petäjä-Repo et al., 2000, 2002, 2006; Cahill et al., 2001a,b; Commons et al., 2001; Wang and Pickel, 2001; Commons, 2003; Guan et al., 2005; Lucido et al., 2005; Gendron et al., 2006). If the hope is to develop pain therapies targeting DOP, strategies to increase its levels of expression at the neuronal plasma membrane need to be described. Ways to control the trafficking of DOP and to increase its cell surface expression include prolonged morphine treatment (or other MOP agonists) or inflammation induced by complete Freund’s adjuvant (CFA; Cahill et al., 2001b, 2003; Morinville et al., 2003; Gendron et al., 2006, 2007a,b). Indeed, following such treatments the density of DOP at the cell surface increases in spinal cord (Cahill et al., 2001b; Morinville et al., 2004), DRG (Cahill et al., 2001a, 2003; Morinville et al., 2003; Gendron et al., 2006, 2007a,b), and central gray neurons (Lucido et al., 2005). As of to date, the mechanisms involved in this process remain poorly described. Several DOP interacting partners have been identified, some of which are involved in protein trafficking. Here, we review these DOP partners in light of their potential role in controlling the cellular trafficking of DOP and, by way of consequence, the analgesic potency of DOP agonists. Depending on their role in regulating the trafficking of DOP toward the plasma membrane, targeting these proteins may serve as a strategy to develop new drugs capable of increasing the analgesic potency of DOP agonists.

There are two major trafficking pathways described for membrane proteins, namely the regulated (or secretory) and the constitutive pathways. Numerous evidence supports the idea that DOP uses both paths to reach the plasma membrane. After being synthesized in the endoplasmic reticulum (ER), membrane proteins undergo a quality check. Improperly folded proteins will be targeted to lysosomes for degradation while those correctly folded will process to the Golgi apparatus where they will undergo post-translational modifications like glycosylation (Sicari et al., 2019). Mature proteins then progress toward the plasma membrane either through the regulated or the constitutive pathway. The protein cofilin, an actin severing protein and a potent regulator of actin filament dynamics, is involved in protein trafficking through the constitutive pathway. Cofilin has a role in improving or repressing the release of proteins from the Golgi apparatus to the cell surface (Heimann et al., 1999; Egea et al., 2006; Salvarezza et al., 2009). Its ability to bind and depolarize actin is, however, inhibited when it is phosphorylated by LIM-kinase 1, a serine/threonine kinase that has LIM and PDZ domains (Yang et al., 1998). This kinase may participate in the cytoskeleton reorganization by phosphorylating cofilin. Cofilin and proteins such as chronophin and LIM kinase interact and colocalize at the membrane level with β-arrestin (β-arr), supporting a role for the latter in regulating their activity (Zoudilova et al., 2007). These processes were recently shown to be involved in the trafficking of DOP to the plasma membrane (Mittal et al., 2013). In this context, strategies to decrease β-arr1 or its interaction with DOP, as well as ROCK or LIM kinase inhibition represent ways to prevent cofilin activation and, by way of consequence, to increase DOP-mediated effects.

An association between DOP and downstream effectors such as the Kir3 ion channel has also been described (Richard-Lalonde et al., 2013; Nagi et al., 2015). BRET and co-immunoprecipitation assays revealed that DOP associates with Kir3 channel subunits in cortical neurons where they also co-internalize upon stimulation with an agonist (Nagi et al., 2015). Other known regulators of DOP trafficking are the G protein-coupled receptor kinases (GRKs). GRKs mediate the phosphorylation on their cytoplasmic tail of numerous GPCRs, including DOP. When phosphorylated by GRKs, DOP promotes the recruitment of β-arrestin, inducing its accumulation into clathrin-coated pits and endocytic vesicles (Chu et al., 1997; Zhang et al., 1999; Whistler et al., 2001). The endocytosis of DOP plays a role in DOP downregulation by triggering its traffic to the degradation path (Law et al., 1984). Although observed in various cellular models and native neurons (Tsao and von Zastrow, 2000; Scherrer et al., 2006), the degradation path is not the only outcome for the sorting of DOP following its internalization. Indeed, the fate of internalized receptors is controlled by the endosomal sorting complex required for transport (escrt). The escrt complex distinguishes the ubiquitinated receptors and guides them to the multivesicular bodies (MVB) where they are addressed to the lysosomes for degradation (Henne et al., 2011). The ubiquitination process is quite essential for the commitment of receptors into a given degradation pathway. For example, the epidermal growth factor receptor (EGFR) ubiquitination is an essential step toward its degradation (Raiborg and Stenmark, 2009). Concerning DOP, ubiquitinated receptors are targeted to MVBs but, in opposition to EGFR, neither its ubiquitination nor its targeting to MVBs are necessary steps for the degradation in lysosomes (Tanowitz and Von Zastrow, 2002; Hislop et al., 2009). Admittedly, any proteins involved in the internalization process and the routing of DOP within the endosomal pathway represent potential targets to alter (either positively or negatively) its functions. Therefore, strategies to increase the density of DOP at the cell surface and its analgesic properties may be directed toward these pathways.

Other actors involved in the regulation of DOP trafficking are the noncanonical signaling proteins and the regulatory proteins. First, calmodulin and periplakin are modulators acting as blockers of DOP. Calmodulin, or calcium-modulated protein, is a widely distributed and versatile member of the calcium-binding protein family (Stevens, 1983). At resting state, calmodulin can constitutively associate with DOP (Wang et al., 1999). Once activated, the binding of calmodulin to DOP is abolished, allowing the receptor to couple to G proteins. The signaling protein periplakin, a member of the plakin family which serves as epidermal cytolinkers and components of cell-cell and cell-matrix adhesion complexes (Aho et al., 2004), interacts with DOP, more specifically with its cytoplasmic tail. The interaction was confirmed using a yeast two-hybrid system, and the site was profiled at the level of residues 321–331, according to the analogy with MOP receptors (Feng et al., 2003). As for calmodulin, periplakin seems to block the G protein activation by competing with its interaction site on the DOP (Wang et al., 1999; Feng et al., 2003). It can be argued that any compound able to selectively block the interaction between DOP and these proteins could increase DOP-mediated effects.

RGS4 is yet another regulator of DOP trafficking. RGS4 interacts with the first 26 amino acids of the C-terminal tail of DOP. Coimmunoprecipitation assays between DOP and RGS4 show that agonist stimulation does not alter the interaction, suggesting that they are constitutively associated (Georgoussi et al., 2006; Wang et al., 2009). However, the distribution of RGS4 is shifted from the cytosol to the membrane upon activation of DOP (Leontiadis et al., 2009). Another protein called spinophilin binds to the same C-terminal region and the third intracellular loop (ICL3) of DOP (Feng et al., 2000). Spinophilin is a ubiquitous multidomain-scaffold protein interacting with actin and protein phosphatase-1 (PP1). Along with RGS4, Gα, and Gβγ subunits, spinophilin forms a complex involving specific regions of the protein and the C-terminal tail of MOP and DOP (Fourla et al., 2012). In HEK293 cells, a constitutive interaction between spinophilin and DOP as well as an altered state following agonist administration was also observed (Fourla et al., 2012). Although the exact role of each association is not yet fully described, their modulation has the potential to regulate the activity of DOP and its downstream effectors and possibly it’s trafficking.

As briefly discussed above, the interaction between GRKs and DOP is well established. As shown by coimmunoprecipitation studies, the association of GRKs with DOP increases following treatment with an agonist. More specifically, GRK2 is translocated to the membrane where it can phosphorylate the C-terminal tail DOP upon stimulation (Li et al., 2003; Zhang et al., 2005). The interaction between GRKs and Gβγ at the membrane is an important step for the translocation of GRK2 from the cytoplasm to the cell membrane (Li et al., 2003). The rapid association of GRK2/GRK3 to DOP following the binding of an agonist is also observed in live cells. The colocalization of GRK2 and DOP is detected in endosomes after 15 min of stimulation suggesting that the complex translocates to clathrin-coated vesicles (Schulz et al., 2002). This appears to be specific to GRK2 and DOP since colocalization is neither detected between DOP and GRK6 nor MOP and GRK2. The interaction between DOP and GRK2 requires the presence of Gβγ subunits (Schulz et al., 2002; Li et al., 2003).

Colocalization and direct protein-protein interaction assays revealed that the recruitment of βarr1 and βarr2 to DOP is induced either by receptor activation (Whistler et al., 2001; Zhang et al., 2005; Qiu et al., 2007; Molinari et al., 2010; Audet et al., 2012) or its phosphorylation by protein kinase C (PKC; Xiang et al., 2001). The interaction sites for both arrestins on DOP are located in the ICL3 (Leu235-Ile259) and the C-terminal tail of DOP (Gln331-Ala372; Cen et al., 2001b). These two regions bind to non-overlapping sites on βarr1 (Cen et al., 2001a). Indeed, a DOP mutant lacking the last 15 residues of the C-terminal tail showed only a reduction in the βarr1 association (Cen et al., 2001a). The ability of a mutant DOP lacking all serine and threonine residues of the C-terminal tail to recruit βarr (Qiu et al., 2007) further supports the contribution of distinct regions.

The interaction between βarr2 and DOP can also occur independently of phosphorylation (Zhang et al., 2005; Qiu et al., 2007). Indeed, both wild-type DOP and a mutant receptor containing a substitution of all C-terminal serine and threonine residues remain capable to recruit βarrs. Instead, an increase in the interaction between DOP and βarr2 can be observed (Qiu et al., 2007). βarr2 plays a major role in the desensitization of DOP (Qiu et al., 2007) while both βarr1 and βarr2 similarly contributes to the internalization process. The link between the fate of post-endocytic receptors and βarrs has also been described. DOP lacking phosphorylation sites in its C-terminal tail is preferentially degraded *via* a βarr2-mediated mechanism. In contrast, a small portion of the wild-type receptor is recycled back to the cell membrane *via* βarrs (Zhang et al., 2008). As mentioned above, interfering with the recruitment of βarrs represents a strategy to increase the functions of DOP, possibly through the inhibition of its internalization and desensitization.

Among DOP-interacting proteins, GASP-1 and the glycoprotein M6a play a crucial role in its regulation. G protein-coupled receptor-associated sorting protein 1 (GASP-1) is a member of the GASP family of proteins. GASP-1 is the most studied member of the family and the only one interacting with DOP (Simonin et al., 2004). This sorting protein is involved in the delivery of receptors to the multivesicular bodies (MVBs; Whistler et al., 2002b; Marley and von Zastrow, 2010). The GASP machinery retains DOP to the endosomes, therefore preventing its recycling (Whistler et al., 2002a; Marley and von Zastrow, 2010; He et al., 2013). This machinery also participates in the transport of internalized DOP to the lysosomes, a process independent of ubiquitination or MVBs. The second interacting protein, the glycoprotein M6a, has been identified using a yeast two-hybrid approach (Wu et al., 2007). M6a is a member of the proteolipid membrane proteins (PLP) family and is primarily expressed in neurons (Yan et al., 1993, 1996; Roussel et al., 1998). It interacts with MOP, affecting its endocytosis and recycling, as well as with other GPCRs, including DOP (Wu et al., 2007). When co-internalizing with DOP, M6a significantly increases its localization within the recycling endosomes, supporting a role for this protein in the post-endocytic sorting and the recycling of receptors (Liang et al., 2008).

An exhaustive study using yeast two-hybrid screening on different C-terminal tails of GPCRs identified four different proteins thought to be involved in the post-endocytic sorting of GPCRs (Heydorn et al., 2004). Two of them being involved in the recycling pathway, ezrin-radixin-moesin-binding phosphoprotein 50 (EBP50, also called Na+/H+-exchanger regulatory factor-1 or NHERF-1) and N-ethylmaleimide-sensitive factor (NSF); while the other two being involved in targeting receptors to lysosomal degradation, GASP-1 and sorting nexin 1 (SNX1). Although the role of NSF and SNX1 in DOP trafficking is yet to be determined, the role of GASP (discussed above) and NHERF-1 are documented. NHERF-1 is a PDZ domain-containing scaffolding protein that has many functions such as protein complex assembly and sorting of internalized GPCRs (like β2-adrenergic and kappa opioid receptors) to the recycling pathway (Huang et al., 2004; Liu-Chen, 2004; Weinman et al., 2006; Hanyaloglu and von Zastrow, 2008). There is also evidence suggesting that DOP interacts with NHERF-1. Indeed, NHERF-1 and DOP can be co-immunoprecipitated from a brainstem extract, an effect increased in morphine-treated animals (Bie et al., 2010). The upregulation of NHERF-1 in transfected cells increases the sorting of DOP through the exocytotic trafficking, improving its membrane insertion and functional expression (Bie et al., 2010). A better knowledge of the mechanisms regulating the association of these proteins with DOP as well as their exact implication in the trafficking of DOP may help to develop drugs aiming at improving the recycling of DOP while reducing its degradation.

More recently, we used mass spectrometry analysis and identified new DOP-interacting partners in transfected HEK293 cells (St-Louis et al., 2017). Among them, we found many subunits of the coatomer protein complex I (COPI). This complex is involved in the transport of proteins from the Golgi to the ER. The interaction between DOP and COPI might explain why DOP is largely retained intracellularly. Using two different subunits of the COPI complex, β-COP and β’-COP, we confirmed the interaction of DOP with the COPI complex. Within its different intracellular loops and C-terminal tail, DOP has 13 putative COPI binding motifs (KxK, RxR, RxK or KxR). Using mutagenesis, we found that the disruption of two motifs, namely K164-K166 (ICL2) and K250-K252 (ICL3), significantly increased the expression of DOP at the surface compared to the wild-type receptor (St-Louis et al., 2017). Shortly afterward, another study described a role for COPI in the regulation of DOP transport to the plasma membrane in neuronal cells (Shiwarski et al., 2019). A conserved COPI binding motifs (RxR) in the C-terminal tail of DOP is indeed required for the adequate delivery of DOP to the plasma membrane. Another key point in the transport of DOP to the cell surface is through its association with a phosphatase and tensin homolog (PTEN). A study visualizing DOP’s trafficking and localization implicated a PTEN-regulated checkpoint in the retention of DOP in primary neuronal cell culture (Shiwarski et al., 2017). After PTEN inhibition, receptors available at the surface are increased, leading to an increase in DOP-mediated antinociception (Shiwarski et al., 2017).

The last partner to be reviewed here is the cyclin-dependent kinase 5 (Cdk5). Cdk5 is a member of the CDK family but unlike the other CDKs, Cdk5 is not involved in the cell cycle progression. This serine/threonine kinase is rather involved in different processes like neuronal activity, neuron migration and neurite outgrowth. It phosphorylates a consensus sequence [(S/T) PX (K/H/R; Beaudette et al., 1993; Songyang et al., 1996)] when activated by its specific neuronal activator, the cyclin-like p35. Such a consensus sequence is present within the second intracellular loop of DOP (T^161^PAK^164^). The threonine (T^161^) residue was previously shown to be phosphorylated by Cdk5 in neuronal cells (Xie et al., 2009). When Cdk5 is inhibited with the CDK inhibitor roscovitine or when a T161A mutant of DOP is used, the level of cell surface DOP is significantly decreased (Xie et al., 2009). A more recent study also supported a role for Cdk5 in the regulation of DOP trafficking. Using the above mentioned Cdk5 inhibitor or by blocking the phosphorylation of DOP by Cdk5 with a mimetic peptide, a decrease in the antinociceptive and anti-hyperalgesic effects of the selective DOP agonist Deltorphin II is also, supporting a lower density of cell surface DOP (Beaudry et al., 2015). Altogether, these observations support a role for Cdk5 in promoting the expression of functional DOP at the cell surface, possibly by promoting its exit from the ER-Golgi.

## Conclusion

DOP represents a promising target for the treatment of pain. As discussed in this review article, the expression of DOP is highly regulated by various mechanisms. In addition to reviewing its distribution along the pain pathways, we discussed how the expression and cellular trafficking of this receptor could be regulated. We focused on mechanisms and protein partners potentially involved in its intracellular retention or its trafficking to the cell surface. What emerges from this review article is the complexity surrounding the regulation of DOP trafficking and functions. As to date, one should admit that the mechanisms involved in DOP trafficking, both under normal and pathological conditions, remain poorly described. A better understanding of the distribution of DOP and how different proteins can affect its signaling and trafficking to the cell surface will facilitate the development of better and possibly less harmful pain therapeutics.

## Author Contributions

BQ, FB, VB and LG wrote the manuscript.

## Conflict of Interest

The authors declare that the research was conducted in the absence of any commercial or financial relationships that could be construed as a potential conflict of interest.

## Footnotes

^1^https://www.drugabuse.gov/drugs-abuse/opioids/opioid-overdose-crisis

^2^https://www.nih.gov/research-training/medical-research-initiatives/heal-initiative/heal-initiative-research-plan
